# 2-(1,3-Benzoxazol-2-ylsulfan­yl)-1-phenyl­ethanone

**DOI:** 10.1107/S1600536809033960

**Published:** 2009-08-29

**Authors:** Hossein Loghmani-Khouzani, Dariush Hajiheidari, Ward T. Robinson, Noorsaadah Abdul Rahman, Reza Kia

**Affiliations:** a Chemistry Department, University of Isfahan, Isfahan 81746-73441, Iran; b Department of Chemistry, University of Malaya, 50603 Kuala Lumpur, Malaysia; c Department of Chemistry, Science and Research Campus, Islamic Azad University, Poonak, Tehran, Iran

## Abstract

In the title compound, C_15_H_11_NO_2_S, a new thio-benzoxazole derivative, the dihedral angle between the benzoxazole ring and the phenyl ring is 9.91 (9)°. An inter­esting feature of the crystal structure is the short C⋯S [3.4858 (17) Å] contact, which is shorter than the sum of the van der Waals radii of these atoms. In the crystal structure, mol­ecules are linked together by zigzag inter­molecular C—H⋯N inter­actions into a column along the *a* axis. The crystal structure is further stabilized by inter­molecular π–π inter­actions [centroid–centroid = 3.8048 (10) Å].

## Related literature

For applications of 2-(benzo[*d*]oxazol-2-ylthio)-1-phenyl­ethanone and β-keto-sulfones in organic synthesis, see: Marco *et al.* (1995 ▶); Fuju *et al.* (1988 ▶); Ni *et al.* (2006 ▶). For uses of haloalkyl sulfones, see: Grossert *et al.* (1984 ▶); Oishi *et al.* (1988 ▶); Antane *et al.* (2004 ▶). For their biological activity, see: Padmavathi *et al.* (2008 ▶). For bond-length data, see: Allen *et al.* (1987 ▶). For hydrogen-bond motifs, see: Bernstein *et al.* (1995 ▶).
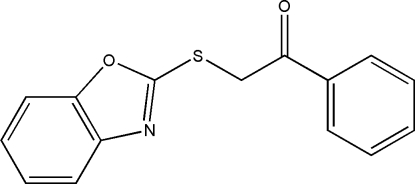


## Experimental

### 

#### Crystal data

C_15_H_11_NO_2_S
*M* *_r_* = 269.31
Orthorhombic, 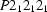

*a* = 4.8580 (2) Å
*b* = 14.0780 (5) Å
*c* = 18.6840 (7) Å
*V* = 1277.82 (8) Å^3^
*Z* = 4
Mo *K*α radiation
μ = 0.25 mm^−1^
*T* = 296 K
0.50 × 0.10 × 0.10 mm

#### Data collection

Bruker SMART APEXII CCD area-detector diffractometer
Absorption correction: multi-scan (**SADABS**; Bruker, 2005 ▶) *T*_min_ = 0.886, *T*_max_ = 0.976
13902 measured reflections
3659 independent reflections
3175 reflections with *I* > 2σ(*I*)
*R*_int_ = 0.037

#### Refinement

*R*[*F* ^2^ > 2σ(*F* ^2^)] = 0.040
*wR*(*F* ^2^) = 0.093
*S* = 1.04
3659 reflections
172 parameters
H-atom parameters constrained
Δρ_max_ = 0.35 e Å^−3^
Δρ_min_ = −0.19 e Å^−3^
Absolute structure: Flack (1983 ▶), 1514 Friedel pairs
Flack parameter: 0.07 (7)

### 

Data collection: *APEX2* (Bruker, 2005 ▶); cell refinement: *SAINT* (Bruker, 2005 ▶); data reduction: *SAINT*; program(s) used to solve structure: *SIR2004* (Burla *et al.*, 2004 ▶); program(s) used to refine structure: *SHELXTL* (Sheldrick, 2008 ▶); molecular graphics: *SHELXTL*; software used to prepare material for publication: *SHELXTL* and *PLATON* (Spek, 2009 ▶).

## Supplementary Material

Crystal structure: contains datablocks global, I. DOI: 10.1107/S1600536809033960/at2859sup1.cif

Structure factors: contains datablocks I. DOI: 10.1107/S1600536809033960/at2859Isup2.hkl

Additional supplementary materials:  crystallographic information; 3D view; checkCIF report

## Figures and Tables

**Table 1:** Hydrogen-bond geometry (Å, °)

*D*—H⋯*A*	*D*—H	H⋯*A*	*D*⋯*A*	*D*—H⋯*A*
C5—H5*A*⋯N1^i^	0.93	2.56	3.395 (2)	149

Symmetry code: (i) 
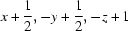
.

**Fig. 1.**
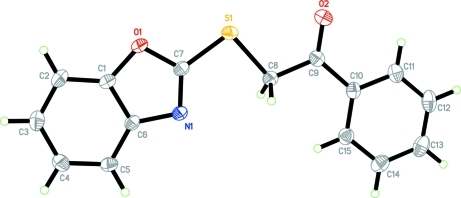
The molecular structure of the title compound, showing 40% probability displacement ellipsoids and the atomic numbering.

**Fig. 2.**
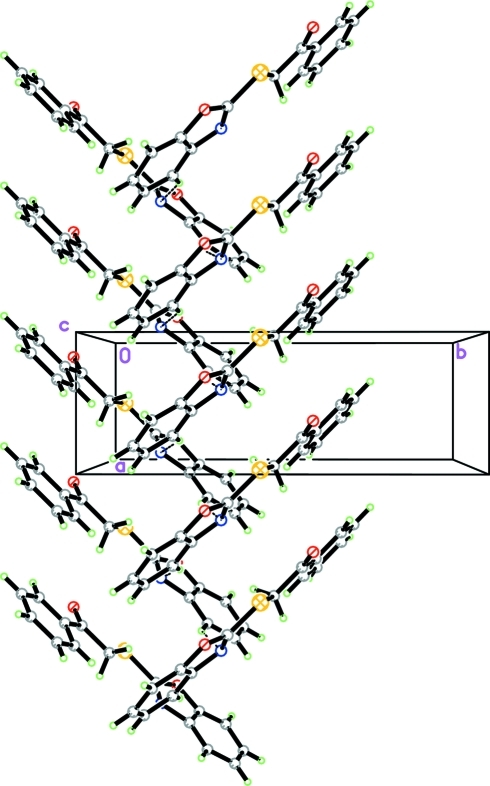
The crystal packing of the title compound, viewed down the c-axis, showing linking of the molecules along the a-axis through intermolecular C—H···N interactions. Intermolecular interactions are drawn as dashed lines.

## supplementary crystallographic information

### Comment

2-(Benzo[d]oxazol-2-ylthio)-1-phenylethanone is of great importance in organic synthesis and β-Keto-sulfones are a very important group of intermediates as they are precursors for Michael and Knoevenagel reactions and are used in the preparation of acetylenes, allenes, chalcones, vinyl sulfones, polyfunctionalized 4H-pyrans and ketones (Marco *et al.*, 1995; Fuju *et al.*, 1988; Ni *et al.*, 2006). In addition, β-keto-sulfones can be converted into optically active β-hydroxy-sulfones, halomethyl sulfones and dihalomethyl sulfones. Halomethyl sulfones and dihalomethyl sulfones are very good α-carbanion stabilizing substituents and precursors for the preparation of alkenes, aziridines, epoxides, and β-hydroxy-sulfones. Haloalkyl sulfones are useful in preventing aquatic organisms from attaching to fishing nets and ship hulls (Grossert *et al.*, 1984; Oishi *et al.*, 1988; Antane *et al.*, 2004). They also possess other biological properties such as herbicidal, bactericidal antifungal and insecticidal. Recently sulfone-linked heterocycles were prepared and have been showed antimicrobial activity (Padmavathi *et al.*, 2008). We prepared this compound as a precursor for synthesis of gem-difluoromethylene- containing heterocycle.

In the molecule of the title compound, (Fig. 1), a new thio-benzoxazole derivative, the dihedral angle between the benzoxazole ring and the phenyl ring is 9.91 (9)°. The interesting feature of the crystal structure is the short C6···S1^i^ [3.4858 (17) Å; (i) -1 + x, y, z] contact which is shorter than the sum of the van der Waals radii of these atoms. In the crystal structure, the molecules are linked together by a *zig-zag* intermolecular C—H···N interactions (Table 1) which packed into a column along the *a* axis (Fig. 2). The crystal structure is further stabilized by the intramolecular π–π interactions [*Cg*1···*Cg*2^i^ = 3.8048 (10) Å].

### Experimental

Sodium carbonate (4.5 mmol) was added to a stirred solution of 2-mercaptobenzoxazole (3 mmol) in ethanol (15 mL) and water (15 mL) and stirred in room temperature for 30 min. α-Bromoacetophenone (3 mmol) was added to the reaction mixture and stirring was continued for 1h. The reaction was monitored by TLC and after 60 min. showed the complete disappearance of starting material. The reaction mixture was poured into 100 mL of 1M HCl containing 50 g of crushed ice. The product was filtered under vacuum and filtrate washed with 10 mL ice-cold ethanol and 10 mL water. Recrystalization from petrol ether and filtration gave the title compound. m.p.: 397-398 K; ^1^H NMR (400 MHz; CDCl_3_): δ 7.86-7.21 (m, 9H), 4.58 (s, 2H). ^13^C NMR (126 MHz; CDCl_3_): δ 194.1 (C═O), 164.3, 148.9, 140.8, 136.1, 132.6, 128.0, 127.8, 124.1, 122.9, 118.3, 109.6, 37.3. IR (KBr, cm^-1^ ): 3027, 2581, 1671 (C═O), 1593, 1492, 1447, 1382, 1326, 1291, 1230, 1182, 1025, 993, 738. Analysis calculated for C_15_H_11_NO_2_S: C 66.89, H 4.12, N 5.20%. Found: C 66.96, H 4.06, N 5.17%.

### Refinement

All of the hydrogen atoms were positioned geometrically [C—H = 0.93–0.97 Å] and refined using a riding model approximation with U_iso_ (H) = 1.2 U_eq_ (C). In the presence of sufficient anomalous scattering the absolute structure was determined (1514 Friedel pairs).

### Crystal data

**Table:**

C_15_H_11_NO_2_S	*D*_x_ = 1.400 Mg m^−^^3^
*M**_r_* = 269.31	Melting point: 398 K
Orthorhombic, *P*2_1_2_1_2_1_	Mo *K*α radiation, λ = 0.71073 Å
Hall symbol: P 2ac 2ab	Cell parameters from 4173 reflections
*a* = 4.8580 (2) Å	θ = 2.6–28.2°
*b* = 14.0780 (5) Å	µ = 0.25 mm^−^^1^
*c* = 18.6840 (7) Å	*T* = 296 K
*V* = 1277.82 (8) Å^3^	Needle, colourless
*Z* = 4	0.50 × 0.10 × 0.10 mm
*F*(000) = 560	

### Data collection

**Table:**

Bruker SMART APEXII CCD area-detector diffractometer	3659 independent reflections
Radiation source: fine-focus sealed tube	3175 reflections with *I* > 2σ(*I*)
graphite	*R*_int_ = 0.037
φ and ω scans	θ_max_ = 30.0°, θ_min_ = 2.6°
Absorption correction: multi-scan (*SADABS*; Bruker, 2005)	*h* = −6→6
*T*_min_ = 0.886, *T*_max_ = 0.976	*k* = −19→19
13902 measured reflections	*l* = −26→26

### Refinement

**Table:**

Refinement on *F*^2^	Secondary atom site location: difference Fourier map
Least-squares matrix: full	Hydrogen site location: inferred from neighbouring sites
*R*[*F*^2^ > 2σ(*F*^2^)] = 0.040	H-atom parameters constrained
*wR*(*F*^2^) = 0.093	*w* = 1/[σ^2^(*F*_o_^2^) + (0.0487*P*)^2^ + 0.1389*P*] where *P* = (*F*_o_^2^ + 2*F*_c_^2^)/3
*S* = 1.04	(Δ/σ)_max_ = 0.001
3659 reflections	Δρ_max_ = 0.35 e Å^−^^3^
172 parameters	Δρ_min_ = −0.19 e Å^−^^3^
0 restraints	Absolute structure: Flack (1983), 1514 Friedel pairs
Primary atom site location: structure-invariant direct methods	Flack parameter: 0.07 (7)

### Special details

**Table:**

Experimental. The low-temperature data was collected with the Oxford Cyrosystem Cobra low-temperature attachment.
Geometry. All esds (except the esd in the dihedral angle between two l.s. planes) are estimated using the full covariance matrix. The cell esds are taken into account individually in the estimation of esds in distances, angles and torsion angles; correlations between esds in cell parameters are only used when they are defined by crystal symmetry. An approximate (isotropic) treatment of cell esds is used for estimating esds involving l.s. planes.
Refinement. Refinement of F^2^ against ALL reflections. The weighted R-factor wR and goodness of fit S are based on F^2^, conventional R-factors R are based on F, with F set to zero for negative F^2^. The threshold expression of F^2^ > 2sigma(F^2^) is used only for calculating R-factors(gt) etc. and is not relevant to the choice of reflections for refinement. R-factors based on F^2^ are statistically about twice as large as those based on F, and R- factors based on ALL data will be even larger.

### Fractional atomic coordinates and isotropic or equivalent isotropic displacement parameters (Å^2^)

**Table:**

	*x*	*y*	*z*	*U*_iso_*/*U*_eq_	
S1	0.01065 (10)	0.44145 (3)	0.66514 (2)	0.02920 (11)	
O1	0.3102 (3)	0.29909 (9)	0.71157 (6)	0.0295 (3)	
N1	0.4022 (3)	0.33643 (10)	0.59627 (7)	0.0250 (3)	
C1	0.5079 (4)	0.23556 (11)	0.68726 (8)	0.0262 (3)	
C2	0.6325 (4)	0.16254 (14)	0.72375 (10)	0.0346 (4)	
H2A	0.5895	0.1480	0.7710	0.042*	
C3	0.8271 (4)	0.11206 (14)	0.68500 (10)	0.0366 (4)	
H3A	0.9193	0.0621	0.7071	0.044*	
C4	0.8889 (4)	0.13370 (13)	0.61400 (10)	0.0341 (4)	
H4A	1.0209	0.0980	0.5900	0.041*	
C5	0.7576 (4)	0.20749 (13)	0.57839 (9)	0.0292 (4)	
H5A	0.7982	0.2218	0.5310	0.035*	
C6	0.5639 (3)	0.25876 (12)	0.61651 (8)	0.0244 (3)	
C7	0.2612 (3)	0.35517 (12)	0.65300 (8)	0.0251 (3)	
C8	−0.0098 (4)	0.47588 (12)	0.57236 (8)	0.0289 (3)	
H8A	0.1664	0.5008	0.5567	0.035*	
H8B	−0.0536	0.4209	0.5432	0.035*	
C9	−0.2291 (3)	0.55059 (12)	0.56314 (9)	0.0266 (3)	
C10	−0.2762 (4)	0.58747 (12)	0.48943 (9)	0.0264 (3)	
C11	−0.4792 (4)	0.65637 (12)	0.47888 (10)	0.0337 (4)	
H11A	−0.5823	0.6780	0.5175	0.040*	
C12	−0.5275 (5)	0.69250 (13)	0.41129 (11)	0.0396 (5)	
H12A	−0.6630	0.7383	0.4045	0.048*	
C13	−0.3744 (4)	0.66049 (14)	0.35363 (11)	0.0397 (5)	
H13A	−0.4070	0.6850	0.3082	0.048*	
C14	−0.1733 (4)	0.59220 (15)	0.36330 (10)	0.0375 (4)	
H14A	−0.0713	0.5707	0.3244	0.045*	
C15	−0.1237 (4)	0.55572 (14)	0.43100 (10)	0.0312 (4)	
H15A	0.0120	0.5099	0.4374	0.037*	
O2	−0.3588 (3)	0.57915 (10)	0.61422 (7)	0.0390 (3)	

### Atomic displacement parameters (Å^2^)

**Table:**

	*U*^11^	*U*^22^	*U*^33^	*U*^12^	*U*^13^	*U*^23^
S1	0.0334 (2)	0.0325 (2)	0.02181 (18)	0.0053 (2)	0.00128 (18)	−0.00418 (15)
O1	0.0362 (7)	0.0345 (6)	0.0179 (5)	0.0024 (5)	0.0020 (5)	0.0012 (5)
N1	0.0279 (7)	0.0290 (7)	0.0180 (6)	0.0018 (6)	−0.0013 (5)	−0.0011 (5)
C1	0.0289 (8)	0.0297 (7)	0.0200 (6)	−0.0035 (8)	0.0007 (7)	0.0003 (5)
C2	0.0439 (11)	0.0348 (9)	0.0253 (8)	−0.0017 (8)	−0.0021 (8)	0.0072 (7)
C3	0.0413 (11)	0.0305 (9)	0.0381 (10)	0.0046 (8)	−0.0074 (8)	0.0052 (8)
C4	0.0343 (9)	0.0313 (9)	0.0367 (10)	0.0049 (8)	0.0024 (8)	−0.0049 (8)
C5	0.0322 (9)	0.0335 (9)	0.0219 (8)	−0.0007 (8)	0.0020 (7)	−0.0034 (7)
C6	0.0283 (9)	0.0263 (8)	0.0185 (7)	−0.0028 (6)	−0.0038 (6)	−0.0009 (6)
C7	0.0276 (8)	0.0279 (8)	0.0198 (7)	−0.0024 (7)	−0.0030 (6)	−0.0012 (6)
C8	0.0290 (8)	0.0332 (8)	0.0243 (7)	0.0037 (8)	0.0008 (8)	0.0017 (6)
C9	0.0253 (8)	0.0227 (7)	0.0316 (8)	−0.0029 (7)	0.0008 (6)	−0.0021 (7)
C10	0.0251 (8)	0.0221 (8)	0.0322 (9)	−0.0040 (6)	−0.0028 (7)	0.0011 (6)
C11	0.0319 (9)	0.0282 (8)	0.0409 (9)	0.0010 (8)	−0.0054 (8)	−0.0018 (7)
C12	0.0377 (11)	0.0300 (9)	0.0512 (11)	0.0011 (9)	−0.0159 (10)	0.0046 (8)
C13	0.0438 (11)	0.0370 (10)	0.0383 (10)	−0.0096 (9)	−0.0134 (8)	0.0083 (8)
C14	0.0359 (10)	0.0449 (11)	0.0317 (9)	−0.0048 (9)	−0.0024 (8)	0.0045 (8)
C15	0.0287 (8)	0.0335 (9)	0.0313 (8)	0.0005 (8)	−0.0023 (7)	0.0035 (8)
O2	0.0440 (8)	0.0376 (7)	0.0355 (7)	0.0095 (6)	0.0076 (6)	−0.0020 (6)

### Geometric parameters (Å, °)

**Table:**

S1—C7	1.7343 (17)	C8—C9	1.507 (2)
S1—C8	1.8028 (16)	C8—H8A	0.9700
O1—C7	1.3704 (19)	C8—H8B	0.9700
O1—C1	1.389 (2)	C9—O2	1.212 (2)
N1—C7	1.290 (2)	C9—C10	1.489 (2)
N1—C6	1.398 (2)	C10—C15	1.393 (3)
C1—C2	1.374 (2)	C10—C11	1.397 (3)
C1—C6	1.389 (2)	C11—C12	1.381 (2)
C2—C3	1.387 (3)	C11—H11A	0.9300
C2—H2A	0.9300	C12—C13	1.385 (3)
C3—C4	1.394 (3)	C12—H12A	0.9300
C3—H3A	0.9300	C13—C14	1.383 (3)
C4—C5	1.389 (3)	C13—H13A	0.9300
C4—H4A	0.9300	C14—C15	1.386 (3)
C5—C6	1.383 (2)	C14—H14A	0.9300
C5—H5A	0.9300	C15—H15A	0.9300
			
C7—S1—C8	95.81 (8)	S1—C8—H8A	109.7
C7—O1—C1	103.29 (12)	C9—C8—H8B	109.7
C7—N1—C6	103.67 (14)	S1—C8—H8B	109.7
C2—C1—O1	128.62 (15)	H8A—C8—H8B	108.2
C2—C1—C6	124.18 (18)	O2—C9—C10	122.17 (16)
O1—C1—C6	107.20 (14)	O2—C9—C8	120.60 (16)
C1—C2—C3	115.13 (17)	C10—C9—C8	117.23 (14)
C1—C2—H2A	122.4	C15—C10—C11	119.18 (16)
C3—C2—H2A	122.4	C15—C10—C9	122.08 (16)
C2—C3—C4	122.13 (18)	C11—C10—C9	118.74 (16)
C2—C3—H3A	118.9	C12—C11—C10	120.30 (18)
C4—C3—H3A	118.9	C12—C11—H11A	119.8
C5—C4—C3	121.36 (18)	C10—C11—H11A	119.9
C5—C4—H4A	119.3	C11—C12—C13	120.01 (18)
C3—C4—H4A	119.3	C11—C12—H12A	120.0
C6—C5—C4	117.13 (16)	C13—C12—H12A	120.0
C6—C5—H5A	121.4	C14—C13—C12	120.29 (18)
C4—C5—H5A	121.4	C14—C13—H13A	119.9
C5—C6—C1	120.06 (16)	C12—C13—H13A	119.9
C5—C6—N1	130.61 (15)	C13—C14—C15	119.98 (19)
C1—C6—N1	109.33 (15)	C13—C14—H14A	120.0
N1—C7—O1	116.50 (15)	C15—C14—H14A	120.0
N1—C7—S1	128.59 (13)	C14—C15—C10	120.25 (17)
O1—C7—S1	114.90 (11)	C14—C15—H15A	119.9
C9—C8—S1	109.67 (12)	C10—C15—H15A	119.9
C9—C8—H8A	109.7		
			
C7—O1—C1—C2	−179.71 (19)	C1—O1—C7—S1	178.11 (12)
C7—O1—C1—C6	0.52 (17)	C8—S1—C7—N1	8.41 (18)
O1—C1—C2—C3	−178.94 (17)	C8—S1—C7—O1	−170.44 (13)
C6—C1—C2—C3	0.8 (3)	C7—S1—C8—C9	177.42 (12)
C1—C2—C3—C4	−0.5 (3)	S1—C8—C9—O2	0.7 (2)
C2—C3—C4—C5	0.0 (3)	S1—C8—C9—C10	−179.75 (13)
C3—C4—C5—C6	0.3 (3)	O2—C9—C10—C15	178.88 (17)
C4—C5—C6—C1	0.0 (2)	C8—C9—C10—C15	−0.7 (2)
C4—C5—C6—N1	179.14 (16)	O2—C9—C10—C11	−0.8 (3)
C2—C1—C6—C5	−0.5 (3)	C8—C9—C10—C11	179.60 (16)
O1—C1—C6—C5	179.26 (15)	C15—C10—C11—C12	0.0 (3)
C2—C1—C6—N1	−179.86 (17)	C9—C10—C11—C12	179.69 (17)
O1—C1—C6—N1	−0.08 (18)	C10—C11—C12—C13	0.0 (3)
C7—N1—C6—C5	−179.68 (17)	C11—C12—C13—C14	0.2 (3)
C7—N1—C6—C1	−0.43 (18)	C12—C13—C14—C15	−0.2 (3)
C6—N1—C7—O1	0.83 (19)	C13—C14—C15—C10	0.1 (3)
C6—N1—C7—S1	−178.00 (13)	C11—C10—C15—C14	0.0 (3)
C1—O1—C7—N1	−0.89 (19)	C9—C10—C15—C14	−179.73 (17)

### Hydrogen-bond geometry (Å, °)

**Table:**

*D*—H···*A*	*D*—H	H···*A*	*D*···*A*	*D*—H···*A*
C5—H5A···N1^i^	0.93	2.56	3.395 (2)	149

Symmetry codes: (i) *x*+1/2, −*y*+1/2, −*z*+1.
